# Safety after BBIBP-CorV (Sinopharm) COVID-19 Vaccine in Adolescents Aged 10–17 Years in Thailand

**DOI:** 10.3390/vaccines10101765

**Published:** 2022-10-21

**Authors:** Saraiorn Thonginnetra, Kriangkrai Tawinprai, Krongkwan Niemsorn, Pathariya Promsena, Manunya Tandhansakul, Naruporn Kasemlawan, Natthanan Ruangkijpaisal, Narin Banomyong, Nanthida Phattraprayoon, Pisuttikan Rangkakulnuwat, Preeda Vanichsetakul, Teerapat Ungtrakul, Kasiruck Wittayasak, Nawarat Thonwirak, Kamonwan Soonklang, Nithi Mahanonda

**Affiliations:** 1 Department of Pediatrics, Chulabhorn Hospital, Chulabhorn Royal Academy, Bangkok 10210, Thailand; 2 Department of Medicine, Chulabhorn Hospital, Chulabhorn Royal Academy, Bangkok 10210, Thailand; 3 Princess Srisavangavadhana College of Medicine, Chulabhorn Royal Academy, Bangkok 10210, Thailand; 4 Chulabhorn Learning and Research Centre, Chulabhorn Royal Academy, Bangkok 10210, Thailand

**Keywords:** COVID-19, SARS-CoV-2, BBIBP-CorV, vaccination, immunization, safety, adolescent, children

## Abstract

Coronavirus disease 2019 affected child health and impacted learning because of the resulting onsite school closures. This prospective cohort study included children aged 10–17 who received two 4 µg doses of BBIBP-CorV administered intramuscularly 21–28 days apart. To assess vaccine safety, 36,808 participants were then followed with paper- and web-based online questionnaire surveys that captured local and systemic reactogenicities following vaccine administration on days 1, 7, and 30. Among participants, 76% (27,880) reported reactogenicity within the first 24 h and 7 days following the first dose. Half (51.41%) of participants experienced pain at the injection site; the majority of cases were mild in severity. Injection site tenderness (37.93%) was another common local reaction. Fatigue (37.89%), myalgia (33.56%), and headache (26.76%) were the most common systemic reactions. On days 2–7 after the first dose, 25.85% of participants experienced adverse reactions. Following the second dose, reactogenicity was 7.6% and 1.09% within 24 h and between days 2–7. The majority of reactions were of mild to moderate severity. We report that two doses of the BBIBP-CorV caused mild to moderate side effects in adolescents in Thailand. The findings confirm the vaccine’s safety profile in this age group.

## 1. Introduction

Infections from severe acute respiratory syndrome coronavirus 2 (SARS-CoV-2) were first discovered in December 2019, and the coronavirus disease 2019 (COVID-19) pandemic has since had a considerable impact on health and the economy worldwide [1,2,3]. The virus is transmitted through aerosols or droplets shed by an infected person [1]. Previous studies have demonstrated the possibility of transmission from asymptomatic or pre-symptomatic patients, making infection control challenging [4,5]. As of late January 2022, the cumulative number of global COVID-19 cases and deaths were approximately 350 million and 5.6 million, respectively [6]. Moreover, the proportion of pediatric patients reported in the United States has continued to increase, from 2% of COVID-19-positive cases at the beginning of the pandemic to 11.5% in late 2020 [7].

COVID-19 has many clinical manifestations that range from asymptomatic infection to life-threatening sickness. Although infections with SARS-CoV-2 in children and adolescents are usually mild or even asymptomatic, children with pre-existing medical conditions are considered to be at risk for severe illness [8]. Furthermore, a rare but severe complication named multisystem inflammatory syndrome in children (MIS-C) has been consistently associated with COVID-19 infection [9].

Despite the availability of several treatment modalities for COVID-19, vaccination plays a central role in disease control [10]. The potential advantages of vaccines include the reduced likelihood of infection in the individual, the prevention of admission to hospital or intensive care, and decreased transmission within a population [11]. COVID-19 vaccines have been developed using a variety of platform technologies including inactivated virus, protein subunit, live-attenuated virus, and virus-vectored and messenger RNA (mRNA)-based technologies [11].

The topic of COVID-19 vaccination in children and adolescents has become unavoidable, particularly in those with underlying medical issues [12]. Vaccination is expected to prevent primary SARS-CoV-2 infection, severe symptoms, and complications such as MIS-C and long COVID-19 syndrome in this age group [13]. Although most children and adolescents with SARS-CoV-2 infection experience mild illness, infection prevention in this cohort is critical for reducing household viral transmission to other vulnerable family members [14]. Vaccinating children and adolescents should also contribute to school reopening and the resumption of outdoor activities [15].

At the study’s start, the BNT162b2 (Pfizer–BioNTech) mRNA COVID-19 vaccine was approved for emergency use in children aged 12 years and older [16]. Unfortunately, a higher incidence of myocarditis was discovered in adolescents—and more commonly in boys—following mRNA COVID-19 vaccination [17].

An inactivated whole virus COVID-19 vaccine, BBIBP-CorV, was developed by China National Biotec Group, Beijing Institute of Biological Products Co., Ltd., Beijing, China [18,19]. BBIBP-CorV was approved by the World Health Organization for emergency use [20]. At the study’s start, China and other countries including Thailand had approved BBIBP-CorV for use in adults 18 years and older [21]. A schedule of two doses of BBIBP-CorV administered by intramuscular injection 21 days apart was used to activate the immune response [19]. With 92–100% seroconversion, BBIBP-CorV is considered to be safe and effective. However, BBIBP-CorV shows a lower T cell reactivity compared with the mRNA vaccine BNT162b2 and the adenoviral vector vaccine ChAdOx1 nCoV-19 (AstraZeneca-Oxford) [22]. A meta-analysis of published articles and real-world data from the Vaccine Adverse Event Reporting System revealed that the inactivated virus caused fewer adverse reactions compared with other COVID vaccine platforms in adult populations [23]. Furthermore, a randomized, double-blind, controlled phase 1/2 trial in China revealed a favorable safety profile and neutralizing antibody geometric mean titer against the SARS-CoV-2 virus among healthy participants aged 18–80 years [24].

In a prospective cohort study of healthy Thai adolescents aged 12–17 years and young adults aged 18–30 years, we observed that immunogenicity in the adolescent group was comparable to that in the young adult group, while BBIBP-CorV caused fewer adverse drug reactions [21]. Therefore, we conducted a clinical trial to evaluate the risk factors, safety, and incidence of SARS-CoV-2 infection after BBIBP-CorV administration in Thai adolescents aged 10–17 years in the broader population. This study further aimed to report the vaccine’s safety profile in particular participants.

## 2. Methods

We conducted a prospective cohort single-center trial at Chulabhorn Hospital, Chulabhorn Royal Academy, Bangkok, Thailand, from 20 September 2021 to 11 November 2021. We enrolled healthy adolescents aged 10–17 years to evaluate the safety and efficacy of the two-dose regimen of BBIBP-CorV. Participants who were pregnant or lactating or had a fever on the day of vaccination were excluded. Participants with a history of SARS-CoV-2 infection, previous COVID-19 or any vaccine in the previous 14 days, upper respiratory tract infection symptoms in the previous 14 days, seizure, abnormal development, or severe allergy to vaccines were also excluded.

Informed consent was obtained from parents and participants before vaccination. The study was conducted in accordance with the guidelines of the Declaration of Helsinki. The study protocol was approved by the Ethics Committee for Human Research, Chulabhorn Research Institute (no. 134/2564) and was registered with thaiclinicaltrials.org (TCTR20210920006).

### 2.1. Interventions

Two doses of 4 µg (0.5 mL) of BBIBP-CorV were intramuscularly administered to all participants with a 21–28-day interval. Participants were observed in the clinic for 15 min following vaccination to detect any immediate side effects.

### 2.2. Safety Measurement

Local and systemic reactogenicities were evaluated using a web-based online survey questionnaire sent by mobile text message on days 1 and 7 post-vaccination for each dose. Pain, tenderness, redness, and swelling were defined as local reactogenicities. Fever, nausea or vomiting, diarrhea, headache, fatigue, and myalgia were defined as systemic reactogenicities.

Local adverse events (AEs) were graded as mild, moderate, and severe if the lesion was less than 5 cm, between 5 to 10 cm, and more than 10 cm, respectively. An AE was considered life-threatening if the participant required an emergency department visit or hospitalization. In addition, systemic AEs were graded mild, moderate, or severe if the AE did not interfere with daily activity, interfered with some daily activity, or limited daily activity, respectively. Systemic AEs were deemed life-threatening if the participant required emergency management or hospitalization in the intensive care unit.

In addition, a 24 h non-questionnaire-based hotline operated by the nurse and pediatric team was available for all participants. Adolescents with side effects were advised by phone and instructed to visit the outpatient clinic or emergency department according to their symptoms. AEs and unsolicited events were followed up over 30 days after the second dose using an online web-based questionnaire survey sent as a mobile text message link.

Before advancement to the second dose, study safety was assessed by a safety monitoring board. All of the unusual events and severe and life-threatening AEs were reviewed.

### 2.3. Outcome

The primary outcome was the safety profile of the BBIBP-CorV vaccine within 7 days of each vaccination and 30 days after the second vaccination. This outcome included the reporting of any unsolicited adverse reactions during this period.

### 2.4. Sample Size

A sample size of 44,554 participants was estimated to provide 80% power and 0.05 type I error based on an anticipated 10% dropout rate and a 3% prevalence of COVID-19 in Thailand when comparing the prevalence of COVID-19 infection among participants and the general population.

### 2.5. Statistical Analysis

Descriptive data were presented as frequency and percentage, and mean and standard deviation. In addition, unsolicited events were presented.

## 3. Results

### 3.1. Participants

A total of 36,808 adolescents between the ages of 10 and 17 who voluntarily enrolled in the study between September and November 2021 were eligible for the study. Participants were scheduled to receive two doses of BBIBP-CorV through intramuscular administration with a 21–28-day interval between doses. Notably, 36,695 individuals (99.69%) received the second vaccination dosage. Of the study dropouts, 14 of 113 were advised by the safety monitoring board not to receive the second dose because of adverse reactions—mostly urticaria and other skin reactions—following immunization.

Initially, we sent text messages to the guardians of the participants asking them to complete the online web-based questionnaire 24 h and 7 days after the first dose administration. However, we received very few responses. Therefore, we used paper-based questionnaires as an alternative, asking participants to complete the questionnaire retroactively when visiting the clinic for the administration of the second vaccine on days 21–28. After the administration of the second dose, we followed up with guardians for 30 days post-vaccination by sending them a link to an online web-based questionnaire survey via telephone text messages. After the first dose, we received 27,880 (75%) responses from the 36,808 participants who completed the survey. Next, 36,695 participants visited the clinic for a second dose and we received 22,224 responses (61%) from this group.

The baseline clinical characteristics of the participants are listed in Table 1. All participants were in a generally good clinical condition.

### 3.2. Safety and Reactogenicity

**Figure 1:**
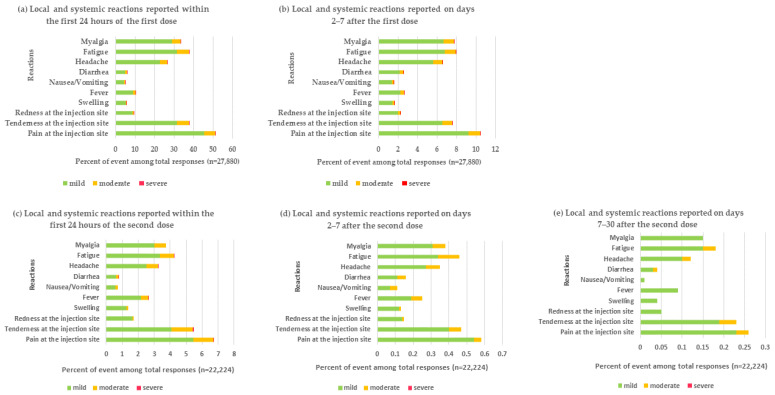
Local and systemic reactions reported within the first 24 h and days 2–7 of the first dose, and within the first 24 h, days 2–7, and days 7–30 of the second dose. Pain, tenderness, redness, and swelling were defined as local reactogenicity. Fever, nausea or vomiting, diarrhea, headache, fatigue, and myalgia were defined as systemic reactions. n: number of participants who responded to the questionnaire.

Overall, 19,537 (70.08%) of 27,880 participants reported reactogenicity within the first 24 h of administration of the first dose of BBIBP-CorV. Half (51.4%) of participants reported injection site pain of mostly mild severity. Another common local reaction was tenderness at the injection site (37.93%). The three most common systemic reactions were fatigue (37.89%), myalgia (33.56%), and headache (26.76%; Figure 1a). On days 2–7 following the administration of the first dose, 25.85% of participants experienced some AEs. Frequent symptoms were similar to those reported in the first 24 h and symptom severity was mild (Figure 1b).

**Figure 2:**
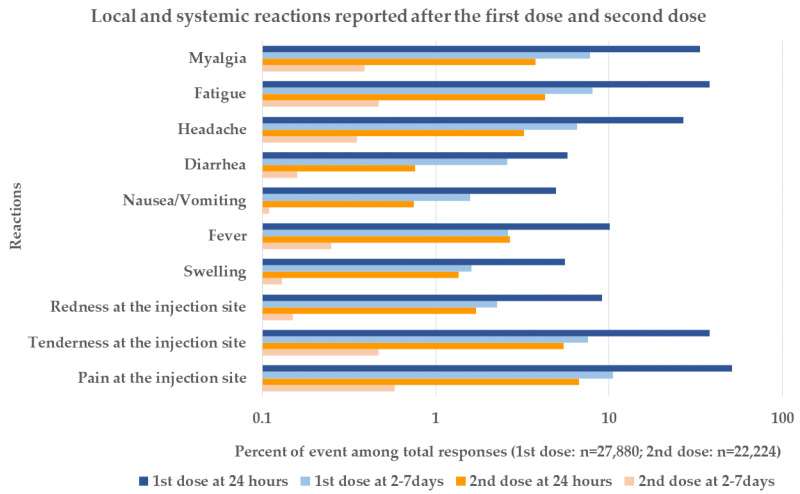
Comparison of the local and systemic reactions reported within the first 24 h and days 2–7 of the first and second dose of BBIBP-CorV in participants aged 10–17 years.

After the administration of the second dose of BBIBP-CorV, 1689 (7.60%) of 22,224 participants reported reactogenicity, which occurred within the first 24 h. The most common symptoms were pain at the injection site (6.74%), tenderness at the injection site (5.49%), and fatigue (4.28%; Figure 1c). In the follow-up on days 2–7, the most common symptoms were similar to those experienced within the first 24 h, but were considerably less frequent (pain at the injection site (0.58%), tenderness at the injection site (0.47%), and fatigue (0.47%); Figure 1d). Most reactions were mild in severity (Figure 1d). The comparison of the local and systemic reactions reported within the first 24 h and days 2–7 of the first and second dose of BBIBP-CorV in participants aged 10–17 years are shown in Figure 2.

Between days 7 and 30 after receiving the second dose of BBIBP-CorV, 83 (0.37%) of 22,224 participants reported local and systemic reactogenicity. The majority of reactions were mild in severity (Figure 1e).

### 3.3. Unsolicited Events

Twelve unsolicited events were reported in eleven cases involving neurological, cardiovascular, severe allergic, or infectious events (Table 2).

#### 3.3.1. Neurological Events

Three neurological AEs were observed following vaccination: left hemiparesis and paresthesia, generalized tonic-clonic (GTC) seizure, and peripheral facial palsy. None of these AEs were life-threatening. The first case was a 13-year-old female adolescent who presented with left hemiparesis and paresthesia approximately 2 h after the first BBIBP-CorV injection. The patient’s neurological examination showed left hemiparesis and decreased sensation in the left side without long tract signs. The motor power was grade IV/V on the left side and grade *v/v* on the right side. The cranial nerves were intact and the results of emergency cranial magnetic resonance imaging were unremarkable. The patient’s signs and symptoms spontaneously resolved after 2 days. Therefore, we suspected immunization-related focal neurological syndrome.

The second case was a previously healthy 14-year-old female with two episodes of GTC seizure. The first seizure episode occurred 17 days after the first vaccination. The patient did not have recurrent seizures and did not take anti-seizure medication. The second dose of BBIBP-CorV was given to the patient 3 weeks after the first dose. The patient suffered a second GTC seizure lasting approximately 5–10 min 4 h after the injection. At the hospital, her physical examination was unremarkable. The patient was diagnosed with epilepsy, given the two episodes of unprovoked GTC seizures. She was treated with the anti-seizure medication phenytoin and was lost to follow-up. The last case was a 14-year-old male who presented with right peripheral facial palsy 5 days after the first vaccination. The patient’s symptoms progressed to complete right peripheral facial palsy within a week. His neurological examination was otherwise unremarkable. The patient was diagnosed with Bell’s palsy, which was confirmed with an electrodiagnostic study. He was treated with oral prednisolone and artificial tears for eye care. The patient further underwent a 10-week rehabilitation program. By the end of the last program session, the patient had completely recovered.

#### 3.3.2. Cardiovascular Events

Four cases reported palpitations after the first dose of BBIBP-CorV. The investigation revealed that one case suffered from sinus tachycardia and the second had premature atrial contraction with self-limited symptoms within one day. The third case was a 10-year-old-female diagnosed with postural orthostatic tachycardia syndrome (POTS) confirmed with a tilt table test. Despite supportive treatment, the patient required beta-blocker therapy to control symptoms and remains on follow-up. The fourth case was a 12-year-old-male who presented with chest pain and light-headedness. The patient was diagnosed with POTS using the active stand test and symptoms resolved within 2 months of starting lifestyle modification.

Two cases of myocarditis were reported after the first dose of BBIBP-CorV; however, only one case may be related to the vaccine. After receiving the vaccine, the 13-year-old male had chest pain on day 5 that lasted for 2 days. A diagnosis of acute myopericarditis was based on clinical symptoms and the elevation of high sensitive cardiac troponin I and creatine phosphokinase-MB while other possible causes were ruled out. Findings from an echocardiogram and cardiac magnetic resonance imaging were unremarkable. The second case suffered from dyspnea from day 1 of receiving the vaccine; however, the onset of illness was reported one day before vaccine administration.

#### 3.3.3. Allergic Reactions

The 13-year-old non-atopic female adolescent with left hemiparesis and paresthesia mentioned above was clinically diagnosed with anaphylaxis. The patient further experienced chest tightness, vomiting, and abdominal pain within 2 h of injection of the first dose of BBIBP-CorV. The patient was treated with intramuscular epinephrine and the anaphylactic symptoms fully resolved within minutes.

#### 3.3.4. Infectious Events

The previously healthy 16-year-old male adolescent presented with painful skin eruption on his right waist and back 5 days after receiving the first dose of BBIBP-CorV. Anti-HIV test samples were non-reactive and Tzanck smear revealed multinucleated large cells. Herpes zoster was the definitive diagnosis.

## 4. Discussion

Vaccination for COVID-19 in children and adolescents helps prevent severe symptoms or hospitalization and serious complications such as MIS-C [25,26]. BBIBP-CorV inactivated vaccine was approved by Chinese authorities for emergency use in children aged over 3 years [27]. In a Thai study, two doses of the BBIBP-CorV inactivated vaccine administered 21 days apart were found to be safe and well-tolerated in adolescents 10–17 years of age [21]. Reactogenicity occurred primarily after the administration of the first dose while local and systemic reactions were mild to moderate in severity [21].

We conducted the study during September to November 2021, in which Thailand had just passed the worst of the fourth wave of the pandemic [28]. Delta variant was the dominant variant, which caused an average of more than 13,000 new cases a day, where Bangkok and its vicinity were the center of the epidemic [28]. During the beginning of the study period, 29.5 million people or 59% of the adult population had received the first dose, and 15 million people (31.5%) had received a second dose of the COVID-19 vaccine [28]. However, there were no approved COVID-19 vaccines for children under 18-year-old available in Thailand at that time. Moreover, parental hesitancy to vaccinate their young children against COVID-19 in Thailand remained high, especially in children younger than the age of 12, which was higher than children over the age of 12 (56.9% vs. 17.1%) [29]. While the study in China has proven the vaccine safety in children over age of 3 [30], thus we chose to conduct a clinical trial in children from 10–17 years of age, aiming to demonstrate the safety profile of the COVID-19 vaccine, and to evaluate the risk factors and incidence of SARS-CoV-2 infection after BBIBP-CorV administration in a younger age group.

Our results support that adverse reactions were transient and mild to moderate in severity; potentially life-threatening AEs were rare among local and systemic reactogenicities (<0.05%). A higher rate of AEs was associated with the first dose, especially 24 h after vaccination. Injection-site pain was the most frequently reported local reaction, while fatigue, myalgia, and headache were the most commonly reported systemic reactions. Our results were similar to those of the phase 1/2 Chinese trial of BBIBP-CorV in participants aged 3–17 years [30]. This study likewise indicated that AEs occurred predominantly after the first dose of vaccination and the most common local and systemic reactions were injection site pain and fever, respectively. The frequency of AEs in our study was higher than that in the previous study of BBIBP-CorV in children [30]. In our study, at least one adverse reaction was reported with a frequency of 71.18% within the first 7 days of the first dose and 8.32% after the second dose, while reports of adverse reactions with BBIBP-CorV in cohorts aged 3–5 years, 6–12 years, 13–17 years, and 18–59 years in the Chinese study were 31.08%, 25.79%, 34.13%, and 23%, respectively [30].

The safety profile of BBIBP-CorV in healthy children and adolescents aged 3–17 years was similar to that of other inactivated vaccines. Most reactions were transient and mild to moderate in severity; fewer than 1% of participants experienced grade 3 adverse reactions. At least one adverse reaction was reported in 27% of participants within 28 days of each vaccination. Injection-site pain and fever were the most frequently reported symptoms [31]. Our findings also have a similar pattern in overall reactogenicity as a systematic review of COVID-19 vaccine‘s safety analysis among children and adolescents [32]. This systematic review included the reactogenicity after receiving BNT162b2, mRNA-1273 (Moderna, Cambridge, MA, USA), BBIBP-CorV, CoronaVac (Sinovac Biotech, Beijing, China), and a recombinant adenovirus type-5 (Ad5)-vectored COVID-19 vaccines, also reported that injection-site pain, fatigue, and headache were the most common adverse events [32]. However, the incidence of reactogenicity after receiving the mRNA vaccine, BNT162b2, was higher [33]. The most common local reaction was injection site pain (86% and 79% after the first and second doses, respectively) [33]. BNT162b2 had a higher rate of reported injection site pain (51.9% and 7.3% after the first and second doses, respectively) compared with that in our study. Systemic reactions to BNT162b2 in adolescent recipients were reported more often after the second dose. Fatigue, headache, and chills were the second most common systemic reactions. Fever (10.3% and 2.9% after the first and second doses, respectively) was less frequently reported in our study of the BBIBP-CorV vaccine, while its incidence was 10% and 20% after the first and second doses, respectively, of the BNT162b2 vaccine [33]. Similar to another inactivated COVID-19 vaccine, CoronaVac, a previous study showed that CoronaVac caused significantly less pain at the injection site than BNT162b2 in both the first (54.5% vs. 89.7%, *p*  <  0.0001) and second vaccination (52.9% vs. 87.9%, *p*  <  0.0001) [34]. Moreover, those who received BNT162b2 reported more other AEs and antipyretics use than CoronaVac [34].

The first of our neurological cases was a female adolescent who presented with left hemiparesis and paresthesia. Her symptoms were comparable to those of an immunization stress-related response called a dissociative neurological symptom reaction. Symptoms including weakness, abnormal movement, or non-epileptic seizures may develop within hours or days after immunization [35]. Clusters of patients experiencing an immunization stress-related response with focal neurological symptoms were observed after the administration of CoronaVac, another inactivated COVID-19 vaccine available in Thailand [36,37]. The median age of study patients was 34 years and sensory loss was the predominant symptom. Most patients reported good recovery. The term “immunization-related focal neurological syndrome” was subsequently proposed for the condition [37]. Therefore, our patient was the first reported case of immunization-related focal neurological syndrome in adolescents. Our second neurological case consisted of a female adolescent with two episodes of GTC seizures after vaccination who was later treated for epilepsy. Seizure is not an uncommon AE following immunization, particularly in cases of a febrile seizure after the measles, mumps, and rubella vaccine [38]. However, a previous study described that in febrile cases, the vaccine merely triggers seizures in patients with underlying genetic or structural defects [39]. In addition, seizures after COVID-19 vaccination commonly occur with other neurological conditions such as cerebral venous sinus thrombosis or vaccine-induced encephalopathy [40]. Given that our patient did not have neurological symptoms other than seizures, we could not conclude that the symptom was vaccine-related.

Our study reported one case of acute facial nerve palsy following the first vaccination dose. This participant responded well to a short course of oral corticosteroids and 10 weeks of facial rehabilitation. Bell’s palsy is a rarely reported AE after COVID-19 vaccination. The case series and nested case-control study in Hong Kong that evaluated the incidence of Bell’s palsy after vaccination estimated a prevalence of 2.0 and 4.8 cases of Bell’s palsy per 100,000 population with the BNT162b2 and CoronaVac vaccines, respectively [41]. This study finding suggested that the CoronaVac vaccine increases the risk of Bell’s palsy with an adjusted odds ratio of 2.385 (95% CI 1.415–4.022). The etiology of Bell’s palsy is unclear. Possible hypotheses include autoimmune disease, inflammation, and viral infection or reactivation [42]. Bell’s palsy after vaccination responds well to oral corticosteroids in adults; however, the evidence in children remains controversial [43,44,45]. The prognosis is good, with a return to normal function within weeks to months [42,46,47].

An increased incidence of myopericarditis after receiving mRNA COVID-19 vaccines has been reported, particularly among young male adults [48,49,50,51]. In a Danish cohort study of BNT162b2, mRNA-1273, ChAdOx1 nCoV-19 (AstraZeneca, Cambridge, UK), and Ad26.COV2.S (Johnson and Johnson, Brunswick, NJ, USA), the incidence of myopericarditis was higher in the mRNA vaccine group than in the non-mRNA vaccine group [52]. Although we observed two potential cases of myopericarditis in our cohort, the first case developed mild symptoms before receiving the first dose of BBIBP-CorV. Thus, vaccine-induced myopericarditis was excluded. The second case was presumed to have vaccine-related myopericarditis as a result of symptoms that developed on day 5 after receiving the first dose of BBIBP-CorV. None of the study participants reported myopericarditis after receiving a second dose of the vaccine. To the best of our knowledge, the case of myopericarditis after the first dose of BBIBP-CorV is the first such case reported in children. The two cases of fulminant myocarditis reported after the administration of the Vero cell type-inactivated COVID-19 vaccine in China were adults aged 57 and 63 years [53]. Although our confirmed case of vaccine-related myopericarditis had mild symptoms and spontaneously and fully recovered after a few days, and despite a systematic review and meta-analysis supporting that the risk of this condition is low [54], close monitoring is suggested.

Interestingly, we observed two cases with a diagnosis of POTS, an orthostatic intolerance condition with multiple etiologies in which the patient develops tachycardia without hypotension while in an upright position [55]. However, this dysautonomia condition has only been reported in patients who have contracted COVID-19 infection [56] or received the mRNA COVID vaccination [57]. Therefore, to the best of our knowledge, these confirmed POTS cases are the first known cases of POTS following the administration of an inactivated vaccine.

We observed one case with clinical symptoms of anaphylaxis. In this case, tryptase was not available because of sample transportation limitations. Severe allergic reaction to the COVID-19 vaccine is rare. The overall incidence rate of anaphylaxis after SAR-CoV-2 administration is 5.00 to 7.91 per million events [58,59]. Given that most COVID-19 vaccine safety studies are based on populations aged 18 to 85 years, data on children are limited. No hypersensitivity reactions were reported in a phase 2/3 trial of BNT162b2 in children aged between 5 to 11 years [60]. The clinical phase 1/2 trial of BBIBP-CorV in participants aged 3–17 years in China revealed only one case of allergic purpura on the second day of the second vaccination dose [30]. The excipient components of COVID-19 vaccines may cause anaphylaxis [61]. Aluminum hydroxide, which is used as an adjuvant, is the main excipient in BBIBP-CorV. After shared decision-making, the participant’s parent declined the second dose of BBIBP-CorV. Interestingly, the rate of anaphylaxis from COVID-19 vaccines is comparable to that of several other vaccines. Furthermore, the anaphylactic reaction rate with COVID-19 vaccines ranks lower than that for rabies, tick-borne encephalitis, measles–mumps–rubella–varicella, and human papillomavirus vaccines, according to the US Vaccine Adverse Event Reporting System and the European EudraVigilance report [62].

Herpes zoster is usually present in elderly or immunocompromised hosts. However, an increasing number of herpes zoster cases have been reported after the administration of the COVID-19 vaccine, even in healthy adults [63]. Interestingly, we report the youngest known adolescent (aged 16 years) who developed herpes zoster 5 days after the first dose of BBIBP-CorV [64].

This study has some limitations. First, the AEs reported after the first dose were retroactively self-reported by participants using paper questionnaires upon visiting the clinic for the second dose. Given that participants retroactively reported their condition in the past 3 weeks, this may have affected information accuracy. We nevertheless received high-quality self-reports via a web-based questionnaire after the second dose, although the response rate was lower after the second dose of vaccination. Second, this study enrolled fewer participants than estimated by sample size calculations because the national vaccine policy authorized the rollout of the competing BNT162b2 vaccine for adolescents while our study was underway. Even though we could enroll only 27,880 participants, our study’s endpoint was to report adverse events. During the data correction period, we found that the maximum adverse events were 51.41%. Therefore, a total of 27,880 respondents had sufficient power, which was 100%. Nevertheless, the study elucidates the safety profile of BBIBP-CorV in adolescents aged 10–17 years in Thailand.

## 5. Conclusions

Two doses of the BBIBP-CorV vaccine were safe and well-tolerated in adolescents. Local and systemic reactions were mild to moderate in severity.

## Figures and Tables

**Table 1:** Demographic characteristics of participants for both vaccine doses.

	Dose 1 N (%)	Dose 2 N (%)
Total participants	27,880	22,224
Sex		
male	14,852 (53.27)	11,717 (52.72)
female	13,028 (46.73)	10,507 (47.28)
Age (mean ± SD *)	13.00 ± 2.33	12.59 ± 2.24

* SD: Standard deviation.

**Table 2:** Unsolicited events within 30 days of the full vaccination procedure.

System	Symptoms/Diseases	Number of Cases
1. Neurological	Transient left hemiparesis and paresthesia/immunization-related focal neurological syndrome	1
	Seizure	1
	Bell’s palsy	1
2. Cardiovascular	Arrhythmia (transient tachycardia and premature atrial contraction)	2
	Postural orthostatic tachycardia syndrome	2
	Myocarditis	1
3. Allergy	Anaphylaxis	1
4. Infection	Herpes Zoster	1

## Data Availability

The datasets generated and analyzed during the study are available from the corresponding author upon reasonable request.
